# Male reproductive tract extracellular vesicles display region-specific heterogeneity in mice

**DOI:** 10.1530/REP-25-0009

**Published:** 2025-06-10

**Authors:** Vipul Batra, Hannah L Morgan, Katie K Choi, David Onion, Nicola Croxall, Kenton P Arkill, James Hallwood, Victoria James, Adam J Watkins

**Affiliations:** ^1^ School of Medicine and Population Health, University of Sheffield, Sheffield, United Kingdom; ^2^ Maternal and Fetal Health Research Centre, Faculty of Biology Medicine and Health, University of Manchester, St Mary’s Hospital, Manchester, United Kingdom; ^3^ School of Medicine, Biodiscovery Institute, University of Nottingham, Nottingham, United Kingdom; ^4^ School of Life Sciences, University of Nottingham, Nottingham, United Kingdom; ^5^ Translational Medical Science, School of Medicine, University of Nottingham, Nottingham, United Kingdom; ^6^ School of Veterinary Medicine and Science, Biodiscovery Institute, University of Nottingham, Nottingham, United Kingdom

**Keywords:** extracellular vesicles, male reproductive tract, epididymosomes, imaging flow cytometry

## Abstract

**In brief:**

Male reproductive tract extracellular vesicles play a critical role in regulating sperm quality and male fertility. This study shows that extracellular vesicles from distinct regions of the male reproductive tract differ in their size, abundance and composition.

**Abstract:**

As sperm transit the male reproductive tract, they undergo a series of dynamic changes, gaining motility, modifying lipid and protein content and refining their epigenetic composition. Extracellular vesicles are central to this post-testicular maturation and changes in their composition could directly impact male reproductive health, sperm quality and post-fertilisation development. This study aimed to characterise and compare extracellular vesicles isolated from distinct regions of the male reproductive tract. Extracellular vesicles were isolated from adult, male C57BL/6J cauda and caput epididymis (epididymosomes) and seminal vesicle fluid by precipitation and size exclusion chromatography. Isolated vesicles were characterised using nanoparticle tracking analysis, transmission electron microscopy, Western blotting and imaging flow cytometry. Epididymosomes and seminal fluid vesicles ranged from 110.26 to 121.26 nm in diameter, had a concentration of 10^9^ to 10^10^ particles/cm^3^ and had a typical round, cup-shaped morphology. The size and concentration of extracellular vesicles from the caput were significantly larger than those from the cauda and seminal fluid. Imaging flow cytometry revealed that all isolated extracellular vesicles expressed CD81 and CD9 tetraspanins; however, CD63 was detected only in caput epididymosomes. Furthermore, there were significantly fewer CD9^+^ vesicles in seminal fluid EVs compared to epididymosomes. Using a range of bulk- and single-vesicle analytical approaches, we show that different regions of the male reproductive tract display distinct vesicle compositional phenotypes. However, additional studies are warranted to define the significance of this heterogeneity, their roles in regulating male reproductive health and the development of their offspring.

## Introduction

The extracellular vesicles (EVs) shed by mammalian cells constitute a cell-to-cell transit system that mediates both short- and long-distance intercellular communication (Yanez-Mo *et al.* 2015, Kalluri & LeBleu 2020, Gurung *et al.* 2021). EVs act as functional vehicles that carry complex and bioactive molecular cargo (including trophic, differentiating, and immune-modulating molecules), which can potentially reprogramme the recipient (target) cell upon interaction by inducing molecular changes in their behaviour, phenotype, physiology and/or function (Kowal *et al.* 2016, Pathan *et al.* 2019, O'Brien *et al.* 2020, Isaac *et al.* 2021).

In the male reproductive tract, post-testicular EVs are secreted by the epididymis, prostate, seminal vesicles, and other accessory glands. Amongst these, the epididymosomes (50–250 nm in diameter) are secreted by the principal cells of the epididymal epithelium into the epididymal lumen (Zhou *et al.* 2018, James *et al.* 2020, Rimmer *et al.* 2021). These epididymal EVs interact with the transiting sperm, inducing significant changes in their protein, sncRNA, glycan and lipid payload composition (Schwarz *et al.* 2013, Trigg *et al.* 2019, Zhou *et al.* 2019, Barrachina *et al.* 2022). The interaction and subsequent incorporation of the epididymosomal cargo into spermatozoa is essential for the development and maturation of sperm. For instance, epididymosomes have been shown to equip the sperm with the necessary components for acquiring motility and the capacity to fertilize the oocyte (Martin-DeLeon 2015, Tecle & Gagneux 2015, Sullivan 2016). Surprisingly, amongst the other male reproductive tract EV subtypes, only prostasomes (secreted from the prostate gland) have been studied in detail. Similar to epididymosomes, prostasomes play vital roles in modulating sperm motility, survival conferring immune protection within the female reproductive tract and facilitating sperm capacitation and acrosome reaction (Park *et al.* 2011, Aalberts *et al.* 2014, Lin *et al.* 2019, Ayaz *et al.* 2021). By comparison, EVs from other accessory glands, e.g. seminal vesicles, are relatively unexplored and their full physiological significance is unknown (Caballero *et al.* 2013, Tamessar *et al.* 2021, Shen *et al.* 2022). The seminal vesicle glands produce most of the ejaculate volume, underscoring their substantial contribution to the composition of seminal plasma (Wang *et al.* 2020). They secrete fructose- and prostaglandin-rich fluid in which the seminal vesicle fluid EVs are suspended (Samanta *et al.* 2018). This fluid also carries enzymes and signalling molecules that influence sperm function and interaction with the female reproductive system (Samanta *et al.* 2018, Wang *et al.* 2020). Despite their significant contribution to semen composition and sperm physiology and function, minimal information exists on phenotypic characteristics, biomolecular payload, and biological functions of seminal vesicle fluid EVs in male reproductive physiology.

At present, most methods for the characterisation of EVs yield data that is averaged across the entire EV population and provide insight into a limited number of parameters (Chiang & Chen 2019). For example, nanoparticle tracking analysis (NTA) used for size and concentration measurements cannot differentiate between EVs and non-EV particles of similar size unless some EV markers are fluorescently labelled. Furthermore, results from techniques which provide an averaged analysis are influenced by the heterogeneous expression of selected markers and signal interference due to free dye molecules or aggregates (Szatanek *et al.* 2017). Likewise, signals produced from western blotting are semi-quantitative and lack information on target heterogeneity in EV populations (Tkach & Thery 2016, Chiang & Chen 2019). Therefore, high-throughput analytical techniques are required, which can provide precise, multiparametric characterization at a single EV resolution to elucidate the inherent heterogeneity in the EVs. Imaging flow cytometry (IFC) is such a method which can differentiate individual EVs from aggregates, debris and noise, providing significantly enhanced resolution and sensitivity when compared to conventional approaches (Gorgens *et al.* 2019, Rees *et al.* 2022). IFC can detect, identify, and characterise individual EVs based on their morphometry, surface protein expression and other EV-associated components resulting in higher specificity and sensitivity (Woud *et al.* 2022). Therefore, in the current study, we sought to characterise EVs isolated from distinct regions of the post-testicular reproductive tract using IFC and conventional analytical methods following the MISEV guidelines and MIFlowCyt-EV framework (Thery *et al.* 2018, Welsh *et al.* 2020, Welsh *et al.* 2024).

## Materials and methods

### Animals

**Figure 1:**
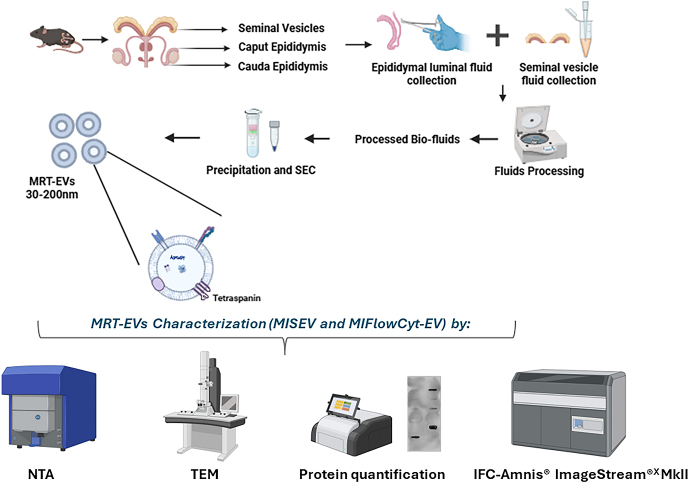
Overall methodology for the characterization of male reproductive tract EVs. Epididymis segments viz. caput and cauda along with seminal vesicles were isolated from adult male C57BL/6J mice and their luminal fluids were processed for the enrichment of EVs by precipitation and SEC. The isolated male reproductive tract EVs (<200 nm) were then characterized by NTA, TEM, total protein quantification (and immunoblotting) analysis and IFC.

All experimental and study procedures were conducted under the United Kingdom Home Office Animal (Scientific Procedures) Act 1986 Amendment Regulations 2012, which transposed Directive 2010/63/EU into UK law, and were approved by the Animal Welfare and Ethical Review Board at the University of Nottingham. Virgin 8-week-old male C57BL/6J mice (Charles River, UK) were maintained at the Bio Support Unit of the University of Nottingham. Males (*n* = 3) were group housed in controlled 12 h light:12 h darkness conditions with a constant temperature (21 ± 3°C) and access to food (standard chow) and water *ad libitum*. Males were culled at 27 weeks of age by cervical dislocation for the collection of caput and cauda epididymides, and seminal vesicles for EV isolation. The overall scheme of work is depicted in Fig. 1.

### Male reproductive tract EVs enrichment

#### Seminal vesicle fluid processing

After culling, both seminal vesicles were excised independently of other accessory glandular tissues and their contents were immediately collected and mixed in 200 μL pre-cooled, particle-free Dulbecco’s phosphate-buffered saline (PBS; Sigma-Aldrich; Cat-D8537, modified, without calcium chloride and magnesium chloride) to reduce coagulation. The collected fluid was centrifuged within 60 min of collection, successively at 300 ***g*** for 10 min, 2,000 ***g*** for 10 min and finally, 17,000 ***g*** for 30 min, each at 4°C to remove the debris and coagulum. The supernatant was snap-frozen and stored at −80°C till further use.

#### Caput and cauda epididymal fluid processing

Caput and cauda epididymides were excised and collected in 1 mL pre-cooled PBS. The caput epididymis was pierced repeatedly using a 26 G needle in 250 μL pre-warmed (37°C) M2 medium (Sigma-Aldrich, UK) on a sterile petri dish under an anatomical microscope. The tissue was gently squeezed using tweezers to extract the epididymal luminal fluid. The tissue and medium were transferred into a 2 mL Eppendorf micro-centrifuge tube and incubated at 37°C for 30 min to allow any remaining fluid to be released from the tissue. The upper 200 μL fluid was transferred to a 1.5 mL Eppendorf micro-centrifuge tube and centrifuged sequentially at 300 ***g*** for 10 min, 3,000 ***g*** for 10 min and finally 17,000 ***g*** for 10 min, each at room temperature. The final supernatant was snap-frozen and stored at −80°C till further use. The cauda epididymis tissue was sliced using ophthalmic scissors in 250 μL pre-warmed (37°C) M2 medium on a sterile petri dish under an anatomical microscope. The tissue, sperm and medium were transferred to a 2 mL Eppendorf micro-centrifuge tube and 250 μL fresh pre-warmed M2 medium was slowly added. The mixture (500 μL) was incubated at 37°C for 30 min to allow the sperm to ‘swim up’. The upper 250 μL fluid containing the motile sperm was transferred to a 1.5 mL Eppendorf micro-centrifuge tube and centrifuged at 400 ***g*** for 10 min at room temperature. The supernatant was removed and subjected to further centrifugation along with the lower fluid fraction (∼250 μL) at 3,000 ***g*** for 10 min, and then at 17,000 ***g*** for 10 min at room temperature to remove any residual debris. The final supernatants were collected, pooled, snap-frozen in liquid Nitrogen, and stored at −80°C before use.

#### EV isolation

The processed epididymal (caput and cauda) and seminal vesicle fluids were thawed on ice and centrifuged at 3,000 ***g*** for 15 min and subsequently at 17,000 ***g*** for 10 min at 4°C. The supernatants were used for isolating EVs using a sequential combination of polymer precipitation-based (ExoQuick® ULTRA EV isolation kit, System Biosciences, UK; Cat-EQULTRA-20A-1) and size exclusion chromatography (SEC)-based (Exo-spin™ SEC columns, Cell guidance systems, UK; Cat-EX03) kits following the manufacturer’s instructions. Briefly, the ExoQuick® precipitation agent was added to processed epididymal luminal or seminal vesicle fluid (1:3.73), mixed well and incubated on ice for 30 min, followed by centrifugation at 3,000 ***g*** for 10 min at 4°C. The supernatant was discarded and the EVs (pellets) were briefly centrifuged to remove the traces of the residual precipitation agent. The pellet was suspended in 200 μL buffer B (proprietary, provided with the kit). This constituted the ‘precipitated’ epididymosomes (EVs derived from caput/caudal epididymis) and seminal vesicle fluid EVs, collectively referred to as male reproductive tract EVs. To remove the non-EV carryover molecules (e.g. proteins, nucleic acids, and lipo-particles) in the precipitated EVs, 200 μL buffer A (proprietary) was added. The mixture was added atop the pre-packed (bipartite resin) purification columns and incubated on a rotating shaker for 5 min. The columns were then transferred to a fresh 2 mL Eppendorf micro-centrifuge tube and centrifuged at 1,000 ***g*** for 30 s at RT to elute the purified, precipitated EVs. For size selection (30–200 nm) and further purification, 100 μL purified, precipitated EVs were applied atop the Exo-spin™ SEC columns (pre-equilibrated with PBS). The liquid was allowed to enter the column matrix under gravity. The SEC columns were then placed into a 1.5 mL Eppendorf micro-centrifuge tube and 180 μL PBS was added as eluent on top. For samples with volume >100 μL, iterative loadings after thorough column flushing (4 × 200 μL) with PBS after every loading were performed (maximum three times) following the manufacturer’s instructions. The final eluates (only the first fraction; particle size: 30–200 nm) were collected and either used immediately or snap-frozen in liquid nitrogen and stored at −80°C till further use.

### Male reproductive tract EV characterization

#### Nanoparticle tracking analysis (NTA)

Male reproductive tract EVs were characterized for size and concentration by Brownian motion analysis using laser scattering microscopy (NTA). The NTA was performed on a ZetaView PMX 120 V4.1 instrument (Particle Metrix GmbH, Germany). A ten-fold dilution series (in PBS) was followed to ensure that fewer than two hundred particles were tracked per image. The instrument was calibrated using a known concentration (1:250,000) of 100 nm polystyrene nanoparticles (Applied Microspheres B.V., The Netherlands). The quality of the cell assembly and particle drift were checked before the size distribution measurements were taken. The default manufacturer software settings were selected for the counting and size measurements of the male reproductive tract EVs and the reference polystyrene nanoparticles. Three cycles were performed by scanning the 11 cell positions for scattering by 40 mW 488 nm laser and capturing 60 frames per second (video setting: high) using the CMOS camera and the following settings: cell temperature- 25°C, camera sensitivity- 80.0 (65 for PS nanoparticles), shutter- 100, gain- 28, tracking radius 2–100 and minimum trace length- 15. After the measurements, the videos and the data were analysed by the in-built ZetaView Software v. 8.02.16 and the graphs for EV size and concentration (median) for each sample group were plotted using MS Excel.

#### Male reproductive tract EVs protein quantification and immunoblotting by Exo-Check™ exosomes antibody arrays

Comprehensive profiling of internal proteins (characteristic EV markers) and multiple EV surface proteins on male reproductive tract EVs was performed using the Exo-Check™ exosomes antibody arrays (System Biosciences, Cat-EXORAY400A). First, the epididymosomes and seminal vesicle fluid EVs were subjected to protein quantification in duplicate using the Pierce micro-BCA protein assay kit (Thermo Scientific, UK) and the low-concentration microplate micro-assay (DC Protein Assay, Bio-Rad, UK), according to the manufacturer’s instructions. The average concentration of the two assays was considered for determining the total protein concentration, which was normalised to total protein concentration (ng)/10^6^ particles. Immunoblotting for the detection of exosome proteins was performed using the mouse Exo-Check™ exosomes antibody (System Biosciences, Cat-EXORAY400A) arrays, following the manufacturer’s instructions. This array contains 12 pre-printed spots, including eight antibodies targeting well-established exosome markers (CD63, CD81, ALIX, FLOT1, ICAM1, EpCAM, ANXA5, and TSG101), along with negative and positive control spots. Briefly, the array membranes were exposed to male reproductive tract EVs-derived protein lysates and were detected using the Pierce™ ECL Western Blotting Substrate (Thermo Scientific™, Cat-32106) on an Odyssey Fc Imaging System (LI-COR).

#### Transmission electron microscopy (TEM)

TEM was used to visualize and characterise the ultrastructure and morphology of the male reproductive tract EVs. The epididymosomes or seminal vesicle fluid EVs suspensions were fixed in freshly prepared 2% formaldehyde (Sigma-Aldrich, UK) and incubated at room temperature for 10 min. Next, amorphous carbon grids (200 mesh; Agar Scientific) were placed onto male reproductive tract EV sample aliquots facing the glow-discharged (non-hydrophobic) side and allowed to settle in a humidification chamber for 30 min. The grids were removed using tweezers, washed twice with 20 μL miliQ water (18.2 MΩ) and dried by blotting with filter paper. For negative staining, TEM grids were further placed onto 10 μL aliquots of 2% aqueous uranyl acetate (0.22 μm filtered) for 1 min at room temperature. The excess solution was blotted carefully without touching the surface of the grid, which was then allowed to air-dry for 10 min. The grids (with EVs) were imaged by TEM (Tecnai T12, FEI) at an accelerating voltage of 100 kV, where transmission electron micrographs were taken at 11kX, 13kX and 18.5kX nominal magnifications using a Gatan Orius Camera with the Gatan Microscopy Suite 3.43 software, giving a scale of 1.76, 2.09 and 2.97 pixels/nm for each of the abovementioned magnifications, respectively. Male reproductive tract EVs diameters were measured in the Fiji ImageJ software to provide an estimate of the size distribution in each subpopulation.

### Imaging flow cytometry (IFC)

#### EV staining using ExoFlow-ONE™ dye and anti-tetraspanin monoclonal antibodies

Intact male reproductive tract EVs were directly detected by the ExoFlow-ONE™ EV Labelling Kit (Emerald Green Gemstone Dye, System Biosciences, Cat-EXOF300A-1) by fluorescently labelling the EV-specific components following the manufacturer’s instructions. Briefly, 250 μL diluted (3X dilution for caput and 2X dilution for cauda epididymosomes and seminal vesicle fluid EVs) samples were resuspended in 500 μL PBS, and 1 μL labelling dye was added to the exosome preparation and incubated at 37°C with shaking for 20 min. For staining with the antibodies for tetraspanins, control titrations were performed for each of the antibodies to determine the ideal concentration, allowing for the most effective differentiation between signal and background, and to establish the optimal incubation protocols. Accordingly, 1 μL anti-tetraspanin monoclonal antibody (CD9- PE/Dazzle™ 594 (BioLegend, Cat-124821), CD63-PE/Cyanine7 (BioLegend, Cat-143909), and CD81-PE (BioLegend, UK; Cat-104905) were added per test (100 μL) conducted. Epididymosomes and seminal vesicle fluid EVs were incubated at 37°C for 30 min. Equivalent concentrations of the isotype controls (ISCs) for the respective antibodies were added to the samples to determine the degree of nonspecific binding. Thereafter, the samples were subjected to SEC to remove any unbound dye and labelled antibodies. An aliquot (50 μL) of the diluted eluates of male reproductive tract EVs (3X dilution for caput and 2X dilution for cauda epididymosomes and seminal vesicle fluid EVs) was used for analysis by IFC on a ImageStream X Mk II imaging flow cytometer. Details on lasers (and channels), acquisition settings, gating, and controls are described in Supplemental Materials and Methods (see section on Supplementary materials given at the end of the article).

### Statistical analysis

Statistical analyses were performed on all quantitative data using the GraphPad Prism 10.3.0 (USA). All data were analysed in triplicates (for *n* = 3 mice/sample) and presented as the mean ± standard deviation (SD). Statistical significance was determined using one-way analysis of variance (ANOVA), followed by Dunnett’s or Tukey’s multiple comparisons post-hoc analysis. TEM data were analysed by one-way repeated measures ANOVA with the Geisser-Greenhouse correction and Holm–Šídák multiple comparisons test. A *P*-value <0.05 was considered statistically significant.

## Results

### Male reproductive tract EVs characterization

**Figure 2:**
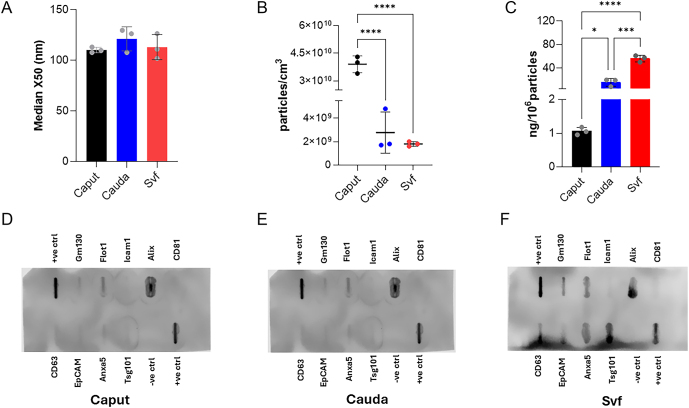
Characterization of male reproductive tract EVs by NTA, protein quantification and Exo-Check™ exosomes antibody arrays. NTA was used to estimate (A) particle size (nm) distribution (median, X50) and (B) concentration (particles/cm^3^) of caput and cauda epididymosomes and seminal vesicles fluid-derived EVs (seminal vesicle fluid EVs). Significantly more particles/mL were detected in the caput vis-à-vis cauda epididymosomes and seminal vesicle fluid EVs samples. (C) Normalized protein content (ng/10^6^ particles) of the cauda epididymosome samples was significantly higher than the cauda epididymosome samples. (D, E, F) Representative blots confirming the presence of exosome markers in the male reproductive tract EV samples. The array was exposed to exosome protein lysates derived from the (D) caput epididymosomes (40 μg), (E) cauda epididymosomes (25 μg), and (F) seminal vesicle fluid EVs (50 μg). The various exosome antibody bands (spots) yielded varying levels of signals depending upon the source of the isolated male reproductive tract EVs. Data are expressed as the mean ± SD. *n* = 3 males per group. One-way ANOVA, Tukey’s post-hoc analyses (*P < 0.05, ***P < 0.001, ****P < 0.0001). Transmembrane or lipid-bound markers – CD63, CD 81, ANXA5, EpCAM and ICAM-1. Cytosolic markers – ALIX, FLOT-1 and TSG101.

The particle enumeration and size distribution analysis of epididymosomes and seminal vesicle fluid EVs samples were first performed by NTA. We observed no significant difference in the average (median) size distribution (X50) of EVs isolated from the caput epididymis, the cauda epididymis or the seminal vesicle fluid (Fig. 2A). The average (median) number of particles/cm^3^ (particles/mL) in the samples from the caput epididymis were significantly higher than the cauda epididymis and the seminal vesicles fluid (Fig. 2B; *P* < 0.0001). Peak analysis (concentration) refers to the point of the frequency distribution that represents the most measured particle size or size range within the sample. This data also indicated an abundance of male reproductive tract EVs in the size range specific to exosomes (<150 nm), which accounted for most particles/cm^3^ across the three male reproductive tract EV populations (Supplementary Table 2).

The total protein equivalent (total protein expressed as ng/10^6^ particles (measured by NTA) differed between the caput and cauda epididymosomes and seminal vesicle fluid EVs. Despite being lower in numbers, cauda epididymosomes carried significantly higher (*P* < 0.05) protein payload (15.71 ng/10^6^ particles) compared to caput epididymosomes (1.06 ng/10^6^ particles) which, however, was significantly lower (*P* < 0.001) than seminal vesicle fluid EVs (56.2 ng/10^6^ particles) (Fig. 2C). We then used the mouse Exo-Check™ exosomes antibody arrays to confirm the presence of EV markers on male reproductive tract EVs. It was observed that these EVs expressed both the transmembrane or lipid-bound markers CD63, ANXA5, and the cytosolic markers ALIX, FLOT-1 and TSG101 (Fig. 2D, E, F).

### Consistent morphological characteristics are observed in EVs across the male reproductive tract

**Figure 3:**
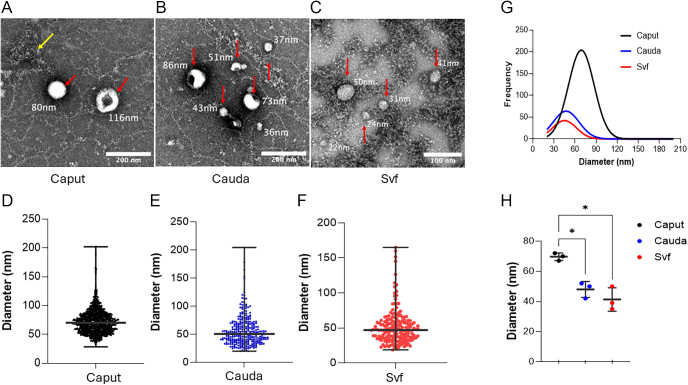
Ultrastructure and morphological characterization of the male reproductive tract EVs by TEM. Representative transmission electron micrographs of the caput (A) and cauda (B) epididymosomes and (C) seminal vesicle fluid EVs indicating the typical spherical, cup-shaped morphology of most EVs. Red arrows indicate EVs with representative measurements and yellow arrows indicate impurities from isolation. The size distribution of the total number of caput (D) and cauda (E) epididymosomes and seminal vesicle fluid EVs (F) with median and range shown. (G) Size distribution of each group fitted to a Gaussian distribution. The peak size frequency for caput epididymosomes was estimated at 70 and 50 nm for cauda and seminal vesicle fluid EVs. (H) Caput epididymosomes were significantly larger than cauda epididymosomes and seminal vesicle fluid EVs. Each dot represents the median diameter for its corresponding biological replicate. Data are expressed as the mean ± SD. The statistical significance was determined by one-way repeated measures ANOVA with Geisser-Greenhouse correction and Holm–Šídák multiple comparisons test (*P < 0.05).

To confirm the ultrastructure and morphology of male reproductive tract EVs, TEM was performed on the negatively stained samples. Most EVs isolated from caput (Fig. 3A) and cauda (Fig. 3B) epididymosomes and seminal vesicle fluid EVs (Fig. 3C) were round, cup-shaped, membrane-enclosed particles consistent with the size and morphology of exosomes (30–150 nm). Despite this, EV measurements from acquired images revealed a differential size distribution (Fig. 3D, E, F) and abundance (Fig. 3G) of the three male reproductive tract EV populations. The median diameter (Fig. 3H) of caput epididymosomes (70 nm) was significantly larger (*P* < 0.05) than the EVs from cauda (51 nm) and seminal vesicle fluid (47 nm).

### Enumeration and characterization of male reproductive tract EVs by IFC

**Figure 4:**
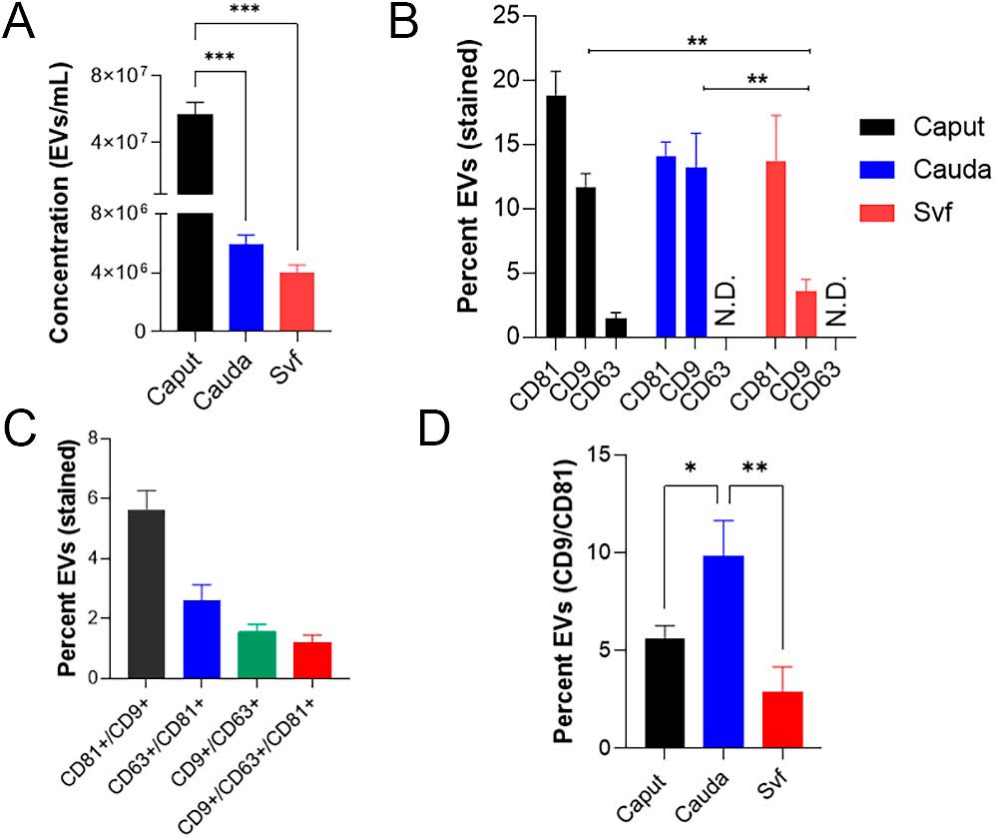
Finding EVs and their immunophenotyping by IFC. ExoFlow-ONE™ labelling (A) revealed a significantly higher concentration of ‘gated’ EVs/mL in caput compared to cauda epididymosomes and seminal vesicle fluid EV samples. (B) Characterization of tetraspanin content of male reproductive tract EVs indicated a distinctive abundance of tetraspanins across the male reproductive tract EV subtypes. Seminal vesicle fluid EVs had significantly fewer percentages of CD9+ vesicles than caput and cauda epididymosomes. (C) Percentage of caput EVs (epididymosomes) expressing either two tetraspanins (CD9+/CD81+ or CD9+/CD63 + or CD63+/CD81+) or expressing all three tetraspanins (CD9+CD63+CD81+) in the whole subpopulation. (D) Percentage of male reproductive tract EVs that stained positively for CD9+/CD81+. Data are expressed as the mean ± SD. One-way ANOVA and unpaired two-tailed *t*-test, Tukey’s post-hoc analyses, *P < 0.05, **P < 0.01 and ***P < 0.001.

The ImageStream X Mk II imaging flow cytometer utilized in this study has successfully been used for EV (including exosomes) detection, enumeration and profiling of multiple tetraspanins and other proteins (Tertel *et al.* 2020, Rees *et al.* 2022). We performed IFC to quantify intact male reproductive tract EVs using the ExoFlow-ONE™ EV labelling dye and determine the percentage of such EVs displaying tetraspanins within each subpopulation of the male reproductive tract EVs (See Supplementary Results for details on calibration, size gating and experimental controls). The number of ‘gated’ EVs/mL in the cauda (5.93 × 10^6^) sample was significantly fewer (*P* < 0.0001) than in the caput (5.65 × 10^7^). Likewise, significantly lesser (*P* < 0.0001) EV numbers were observed in the seminal vesicle fluid (3.96 × 10^6^) compared to the caput epididymis samples (Fig. 4A). These data corroborated the general trends in size distribution profiles and peak analysis (concentration) data generated from the NTA and TEM experiments. Notably, the number of ‘gated’ EVs/mL estimated by IFC (Fig. 4A) and the peak analysis (concentration) data generated from the NTA (Supplementary Table 2) were in consonance. Besides, all the three characterization methods viz. NTA, TEM and IFC indicated the highest abundance of EVs in caput epididymis samples (Figs 2B, 3G, 4A and Supplementary Table 2).

### IFC reveals heterogeneity in male reproductive tract EVs surface expression of tetraspanins

The details regarding population gating and corresponding isotype controls are mentioned in Supplementary Results. Immunophenotyping of male reproductive tract EVs by IFC indicated that seminal vesicle fluid EVs had a considerably lower abundance of CD9+ vesicles than both caput (*P* < 0.01) and cauda (*P* < 0.01) epididymosomes, while the CD81+ vesicle numbers did not vary significantly between the samples (Fig. 4B). The expression of tetraspanin CD63 could only be validated in the caput epididymosomes. In the cauda epididymosome and seminal vesicle fluid EVs samples, a significantly higher binding of isotype control (ISC) for CD63 (*P* < 0.05 and *P* < 0.01, respectively) than the caput epididymosome sample was observed (Supplementary Results). The data for CD63 profiling in the cauda epididymosomes and seminal vesicle fluid EVs were thus excluded because of the high binding of ISCs (>10%) despite multiple standardization steps. Next, we determined the percentage of male reproductive tract EVs expressing either two tetraspanins (CD9+/CD81+ or CD9+/CD63 + or CD63+/CD81+) or expressing all three tetraspanins (CD9+CD63+CD81+). These data were computed for caput epididymosomes (Fig. 4C), which indicated higher abundance of CD9+/CD81+ EVs (since CD63 was excluded from the analyses, the percentage could not be determined for EVs from cauda and seminal vesicle fluid). Notably, the expression of such EVs was significantly higher in cauda compared to caput (*P* < 0.05) and seminal vesicle fluid EV (*P* < 0.01) samples (Fig. 4D).

## Discussion

EVs have gained considerable recognition as key mechanistic factors in the regulation of gamete quality and seminal plasma composition. However, inconsistent findings on the structure, composition and functions of various male reproductive tract EV subtypes indicate that these vesicles are still not well-characterized (Roca *et al.* 2022, Martinez-Diaz *et al.* 2024*a*). This is mainly due to the scarcity of sensitive, high throughput methods to detect and differentiate EVs from non-EV components (debris and noise) at single vesicle resolution and lack of specificity (Roca *et al.* 2022, Barranco *et al.* 2024, Martinez-Diaz *et al.* 2024*a*). We employed both bulk- and single-EV (IFC) enumeration and multiparametric characterization of caput and cauda epididymosomes and seminal vesicle fluid EVs (male reproductive tract EVs). Our results revealed that EVs from different regions of the male reproductive tract in mice display significant heterogeneity in their size, abundance, and immunophenotype profiles. Our data also indicate that IFC can be used as an efficient technology capable of high-throughput identification, enumeration, differentiation, and multiparametric characterization of various male reproductive tract EV subtypes at an individual vesicle level.

We performed characterization of epididymosomes and seminal vesicle fluid EVs according to the MISEV 2018 and 2023 guidelines (Thery *et al.* 2018, Welsh *et al.* 2024), which confirmed the presence of EVs across the three male reproductive tract regions. The interactions of the sperm with the surrounding medium of the distinct epididymal regions are believed to conclude the final steps of spermatogenesis. The molecular constitution of the spermatozoa changes continuously and progressively in the epididymis via extrinsic factors that the sperm encounter within the luminal microenvironment of the epididymal tubule (Tecle & Gagneux 2015, Zhou *et al.* 2018, Zhou *et al.* 2019). Epididymosomes are the key elements of the epididymal luminal milieu, which carry the developmentally important ncRNAs, fertility-modulating proteins, information-rich lipids and glycans, the composition of which changes in a region-specific manner (Martin-DeLeon 2015, Reilly *et al.* 2016, Zhou *et al.* 2019). Emerging evidence suggests that the biomolecular cargo carried by the male reproductive tract EVs is transferred to the transiting sperm, thereby modulating their composition and function (Sullivan 2015, Reilly *et al.* 2016, Nixon *et al.* 2019, Barrachina *et al.* 2022). We observed differences in the number of EVs and size distribution patterns specific to the location (segment) of the male reproductive tract. The generation (and release) of EVs and their cargo contents reflect their source cell type and physiological state. This holds crucial information to reprogramme the target cells or their specific functional regions as a part of a system that enables intra- and intercellular communication (Girouard *et al.* 2009, van Niel *et al.* 2018). If this holds for most EVs, then numeric, phenotypic, and compositional differences between male reproductive tract EVs should also exist (Ayaz *et al.* 2021). Both NTA and TEM discerned numerical and size differences between caput and cauda epididymosomes and seminal vesicle fluid EVs and exhibited similar trends, i.e. caput EVs being the most concentrated and largest. Despite that, TEM and NTA did not reflect identical quantitative data. This may be because NTA signal is less reliable for very small EVs owing to the differences in Brownian motion. Particularly, it fails to report a peak EV diameter below 60 nm (Bachurski *et al.* 2019). Besides, it is very sensitive to temperature and dependent on software settings used for data acquisition that can significantly impact size analysis (Filipe *et al.* 2010, Vestad *et al.* 2017). Moreover, like most bulk EV analysis techniques, NTA cannot distinguish between actual EVs and non-EV particles with similar optical or size attributes of EVs or background signal (noise) (Snyder *et al.* 2021). TEM on the other hand misrepresents the sizing of the EVs causing shrinkage, may fail to represent the complete size spectrum of EVs and the output is protocol- and operator-dependent (Bachurski *et al.* 2019, Rikkert *et al.* 2019). Therefore, complementary characterization of EVs by alternative methods including immune or western blotting and flow cytometry is strongly recommended (Thery *et al.* 2018, Welsh *et al.* 2020, Welsh *et al.* 2024). The same was followed in the current study. The discrepancy in the sizing data for male reproductive tract EVs by TEM and NTA, however, may thus be ascribed to differences in the detection principles of these two complementary yet different techniques.

Interestingly, the biomolecular composition of the male reproductive tract EVs, particularly, their sncRNA and proteomic payloads, vary across the male reproductive tract, indicating their interactional and functional diversity (Girouard *et al.* 2011, Reilly *et al.* 2016, Conine *et al.* 2018, Nixon *et al.* 2019). These EVs selectively transfer a unique set of molecules to the maturing spermatozoa by interacting with them in a region-specific manner (Caballero *et al.* 2013). Besides, the existence of compositional heterogeneity within the specific male reproductive tract regions (e.g. cauda epididymis) has also been reported (Frenette *et al.* 2010). Nonetheless, further studies are warranted to elucidate the molecular cargo carried by the male reproductive tract EVs and their effect on sperm, development and lifetime offspring health and wellbeing. Overall, the heterogeneous male reproductive tract EVs thus may provide a mechanism for selective and bulk delivery of bioactive cargo to the maturing spermatozoa, as a result of which they acquire motility and ability to fertilize the egg.

Identifying the tetraspanins is a routine part of characterizing EVs, including the male reproductive tract EVs (Jankovicova *et al.* 2020). The tetraspanins of epididymosomes are known to be implicated in the interaction and membrane fusion of these EVs with spermatozoa (Sullivan 2016). We employed IFC as a high-throughput alternative technique to western blotting for identifying and quantifying proteins (including tetraspanins) in individual male reproductive tract EVs (Barranco *et al.* 2019, Gorgens *et al.* 2019, Barranco *et al.* 2024). Unlike western blot, which averages results across the populations, IFC can provide insights into EV heterogeneity, requires less sample material, and allows for simultaneous detection of multiple EV markers, offering quantitative, high-throughput data (Tkach & Thery 2016, Morales-Kastresana *et al.* 2017, Chiang & Chen 2019). We used the hallmark EV (exosome) markers CD9, CD63 and CD81 (tetraspanins) for immunophenotyping the male reproductive tract EVs (Jankovicova *et al.* 2020, Tamessar *et al.* 2021) by IFC following the MIFlowCyt guidelines (Welsh *et al.* 2020), which confirmed distinctive immunophenotypic signatures of the male reproductive tract EV subtypes suggesting distinct cellular origins (Marchisio *et al.* 2020, Garcia-Martin *et al.* 2022). As mentioned earlier, the tetraspanins present on the EV surface are also associated with cell recognition and thus can define the interactions of EVs with the specific recipient cells or their specific functional structures or regions. For example, EVs isolated from different male reproductive tract segments interact with particular and distinct sperm functional structures or regions (e.g. detergent-resistant membrane domain) or regions, e.g. head, acrosome or tail (Girouard *et al.* 2009, Andreu & Yanez-Mo 2014, Choy *et al.* 2022).

A higher relative percentage of the CD81+ and CD9+ EVs (compared to CD63+ EVs) has been reported among the porcine seminal fluid EVs and EVs from human semen (Barranco *et al.* 2019, Luo *et al.* 2024). We also observed similar distribution patterns of CD81+ and CD9+ vesicles amongst the three male reproductive tract EV subtypes. The distribution of these vesicles is known to vary throughout the epididymis and subsequent segments of the male reproductive tract (Jankovicova *et al.* 2020, Jangid *et al.* 2023). Contrary to the previous reports on porcine and bovine seminal EVs where CD9+ vesicles are most abundant (Barranco *et al.* 2019, Jankovicova *et al.* 2020, Jangid *et al.* 2023), we observed that the CD81+ vesicles were the most abundant, particularly in EVs collected from the caput epididymis and seminal vesicles fluid. Thus, CD81 not CD9 was the predominant marker in these mice EV subtypes. This discrepancy may reflect differences in species, sample source, or both; however, further investigation is needed to confirm this variation. The CD9+ epididymosomes are implicated in the transferring of necessary biomolecular cargo to sperm during epididymal maturation (Caballero *et al.* 2013, Sullivan 2016, Tamessar *et al.* 2021). The CD9+ epididymosomes isolated from bull epididymis were reported to be enriched in proteins involved in sperm maturation and sperm–egg interaction (Caballero *et al.* 2013). Contrarily, the CD9 epididymosomes reportedly contain a high level of epididymal sperm-binding protein 1 (ELSPBP1) that is specifically transferred to dead spermatozoa in the epididymis and further protects the surviving male gametes (Sullivan 2015, 2016). A recent systematic review of the male reproductive tract EVs isolated from the seminal fluid indicated that the size, protein markers and capacity to interact with sperm varies between fertile males and those with fertility disorders (Sullivan 2015, 2016, Parra *et al.* 2023). The biomolecular payload from these male reproductive tract EVs was implicated in sperm fertilizing capacity, embryo development, and implantation, suggestive of their role in the regulation of male fertility and offspring development (Sullivan 2015, 2016, Parra *et al.* 2023). The composition of the EVs and their associated tetraspanins profile are thus posited to depend on the cellular origin and function and any changes would indicate differences in the biological function of the EV subtypes (Willms *et al.* 2018). It is thus possible that the identified male reproductive tract EV subtypes in this study have distinctive biomolecular cargo and specific roles in male fertility and reproductive outcomes. However, further studies are warranted to validate this.

Interestingly, both CD81 and CD9 molecules have also been detected on the surface of sperm and are suggested to participate in sperm–egg membrane fusion (Jankovicova *et al.* 2016, Frolikova *et al.* 2018). CD63 has also been identified in the sperm equatorial region (associated with gamete interaction) and seminal plasma EVs, suggestive of their role in mammalian fertilization (Alvarez-Rodriguez *et al.* 2019, Jankovicova *et al.* 2020). CD63, although a characteristic EV marker, has previously been reported to be absent in some classes of exosomes, e.g. from B-cells, which, however, express CD9 and C81 (Saunderson *et al.* 2008). Although CD63 epididymosomes have recently been reported to improve sperm function, their expression was detected only in a restricted EV subpopulation of seminal fluid in both humans and animals (Barranco *et al.* 2019, Luo *et al.* 2024). We also could not determine the surface expression of CD63 on cauda epididymosomes and seminal vesicle fluid EVs. The latter are a distinctive class, which, for example, in humans, differs from prostasomes since they lack characteristic CD markers, CD10, CD13, and CD26 and being smaller (Sahlen *et al.* 2010). We also observed that seminal vesicle fluid EVs were smaller and had significantly lesser CD9+ vesicles compared to caput and cauda epididymosomes, thus being different than epididymal EVs. However, akin to epididymosomes, the maximum proportion of these EVs expressed the CD81 tetraspanin. As previously mentioned, porcine seminal plasma-derived small EVs have highest proportion of EVs expressing CD81 (Barranco *et al.* 2019). It is known that seminal vesicles may contribute up to 70% of the ejaculate, making the bulk of seminal plasma, while the epididymal and prostate contributions are much smaller (Yuruk *et al.* 2017, Wang *et al.* 2020). This may explain much higher proportion of EVs expressing CD81 in seminal vesicles fluid compared to epididymosomes. CD81 is functionally implicated in T cell–B cell collaboration inducing T-dependent B cell-mediated immune responses (Mittelbrunn *et al.* 2002). It is not known if CD81+ vesicles regulate the immune responses elicited in female reproductive tract upon exposure to sperm and seminal plasma components including seminal vesicle fluid EVs expressing CD81. Functionally, the interactions between the male reproductive tract EVs and sperm could also be regulated by the presence of different functional membrane domains. For example, both raft and non-raft domains have been demonstrated among the cauda epididymosomes (Girouard *et al.* 2009). Whether the tetraspanins are associated with compartment-specific domains on the sperm surface is not currently known. Nevertheless, compositional and phenotypic heterogeneity exists amongst the male reproductive tract EVs, particularly those involved in regulating sperm physiology (Martinez-Diaz *et al.* 2024*b*).

One of the key limitations is the inability to accurately characterize the size of very small EVs, despite employing advanced high-sensitivity flow cytometers such as the ImageStream X Mk II along with NTA and TEM. Whilst the NTA tends to misrepresent smaller EVs (<60 nm), TEM misrepresents larger EV subtypes because of repeated wash steps during sample preparation (Bachurski *et al.* 2019). Contrarily, the IFC limitations arise from the lack of reliable calibration of the side scattering sensitivity of the instrument, which was benchmarked by measuring the smallest reference bead distinguishable from noise, 110 nm, in this study. Nonetheless, the ImageStream X Mk II imaging flow cytometer can still detect particles much smaller, given they are sufficiently brightly labelled (e.g. ExoFlow-ONE™ labelled), as observed in this study (Gorgens *et al.* 2019, Woud *et al.* 2022). Particularly, the size range of secreted exosomes, including male reproductive tract EVs, typically spans 30–150 nm and carry crucial biomolecular payloads (Sullivan 2016, Willms *et al.* 2018, Rimmer *et al.* 2021, Parra *et al.* 2023, 2024). Previous studies have highlighted the structural and morphological heterogeneity of porcine seminal plasma EVs, reflecting the diverse reproductive organ origins. Notably, smaller EVs (<200 nm) are reported to be more abundant in the seminal plasma fractions from the epididymis and prostate (Parra *et al.* 2024). The composition and putatively the biological roles of small and large EV subsets in seminal plasma are different, thus indicating distinctive biogenesis and functionality (Martinez-Diaz *et al.* 2024*b*). For example, it has been reported that the larger EVs (mean diameter: 303.9 ± 15.86 nm) more effectively modulate the expression of steroidogenesis-related genes in cumulus cells than smaller (mean diameter: 118.4 ± 8.99 nm) EVs (Mateo-Otero *et al.* 2022). Despite the technical limitations associated with IFC, our findings provide valuable insights, particularly the novel identification of distinct tetraspanins immunotype profiles across the three male reproductive tract EV subtypes. This represents the first demonstration of such profiles in the male reproductive tract EVs, including epididymosomes and seminal vesicle fluid EVs, using IFC.

Another technical limitation of this study arises due to the highly viscous nature of seminal vesicles fluid, which makes it particularly challenging to work with. Despite taking measures to reduce coagulation, this fluid retains its viscous characteristics and coagulum formation could not be avoided. This may lead to co-isolation of non-EV contaminants and loss of EVs, possibly explaining the lesser number of EVs identified in seminal vesicles fluid. Future studies using additional purification strategies, such as density gradient ultracentrifugation, could further assess the presence of contaminants such as lipoproteins and refine male reproductive tract EV isolation. Overall, the distinct expression of individual tetraspanins on the male reproductive tract EVs surface may indicate their specific roles in the male reproductive tract, fertilization and beyond (Rimmer *et al.* 2021, Tamessar *et al.* 2021). Further studies elucidating their biomolecular payload are warranted to understand their role in sperm maturation and shaping its epigenome, which can potentially influence the post-mating female reproductive tract physiology and affect the developmental trajectory of the offspring.

## Conclusion

This study highlights the variations in the numbers and immunophenotype profiles of the male reproductive tract EVs collected from the caput and cauda epididymis and seminal vesicles. We demonstrated the effectiveness of high-throughput IFC for detecting, enumerating, and differentiating male reproductive tract EV immunophenotypes. Given their distinct cellular origins, the male reproductive tract EV subtypes may carry differential biomolecular payloads, have distinct functions, and target either specific functional structures of the sperm (epididymosomes) or cells in the female reproductive tract (seminal vesicle fluid EVs) or both. Further research, e.g. comprehensive ‘omics studies’, on the biochemical composition of the payload is necessary to fully understand their role in male reproductive physiology and offspring health.

## Supplementary materials



## Declaration of interest

The authors declare that there is no conflict of interest that could be perceived as prejudicing the impartiality of the work reported.

## Funding

This work was funded by a Biotechnology and Biological Sciences Research Councilhttps://doi.org/10.13039/501100000268 (BBSRC) grant (BB/V006711/1) to A J W.

## Author contribution statement

Conceptualization was performed by A J W and V B. Methodology was given by V B, D O, N C and A J W. V B and A J W helped in investigation. Data curation was performed by V B, K K C, J H, K A, V J and A J W. Formal analysis was done by V B and A J W. A J W helped with funding acquisition. Writing of the original draft was done by V B. Writing of the review and editing was done by V B, H L M, K K C, D O and A J W. All authors have contributed to and agreed to the published version of the manuscript.

## Data availability

All data generated or analysed during this study are included in this published article and its supplementary information files.
