# Supplementary material for: Male reproductive tract extracellular vesicles display region-specific heterogeneity in mice
Source: Reproduction. 2025 Jun 10;170(1):e250009. doi: 10.1530/REP-25-0009 (PMC12152732; doi:10.1530/REP-25-0009)
Supplement: Supplementary file 1 [file supplementary_materials.pdf]

Supplementary Table 1: Assay controls for IFC

Assay Control	Details
Buffer unstained	PBS only with no ExoFlow-ONE™ EV labelling dye or tetraspanins
Buffer stained	PBS and ExoFlow-ONE™ EV Labelling dye
EVs unstained	Epididymosomes and Svf-EVs only with no ExoFlow-ONE™ EV Labelling dye or tetraspanins
Isotype controls	Isotypes for anti-tetraspanins monoclonal antibodies and ExoFlow-ONE™ EV Labelling dye added
Size reference nanobeads	Beads containing diameters of 180nm, 240 nm, 300 nm, 590 nm, 880 nm, and 1300 nm diameter with refractive index η=1.43, and 110nm and 500nm green, fluorescent (blue laser) beads with refractive index η=1.59

Supplementary Table 2: Peak Analysis (concentration) data, also known as mode, indicated the size and abundance of male reproductive tract EVs were consistent with that of exosomes. Data represented as mean from n=3 caput, cauda and seminal vesicle fluid (SvF) replicates for each sample, each from a separate male.

	**Peak Analysis (concentration)**			
	**Diameter (nm)**	**Particles/mL**	**FWHM (nm)**	**Percentage**
**Caput**	103.56	4433333	76.36	100
**Cauda**	110.00	2966666	81.43	100
**Svf**	111.54	1800000	82.12	100

Supplementary Materials and Methods

Imaging flow cytometry (IFC)

EV staining using ExoFlow-ONE™ dye and anti-tetraspanin monoclonal antibodies. IFC was performed on MRT-EVs using an ImageStream X Mk II imaging flow cytometer (Cytek Biosciences) with the following settings: Lasers: 488 nm (power: 200 mW), 561 nm (power: 200 mW) 642 nm (150 mW) and 785 nm (40 mW). A high numerical aperture (0.9) objective lens (60X) with a low acquisition rate was used with Brightfield (BF) set to channels 01 and 09 and side scatter (SSC) to channel 12. Channels 04, 03, and 06 were used for PE/Dazzle™ 594 (CD9), PE (CD81), and PE/Cyanine7 (CD63), respectively. A minimum of 10,000 EV events were acquired (else the acquisition was run for 4 minutes if EV events were <10, 000) using the INSPIRE software. The data acquisition software (INSPIRE) employed in this study enables extensive calibration and self-check tests during the initialisation start-up process resulting in excellent data reproducibility (Rees, et al.

2022). Reference size nanobeads (Apogee Flow Systems) covering sizes of 110-1300 nm, were analysed to assess the light scatter characteristics and fluorescence performance and to display the reference standard. Data analysis was performed using IDEAS software (ImageStream Data Exploration and Analysis, ver. 6.2, Cytek Biosciences). The IDEAS software feature “objects/mL” was utilized to determine the concentration of gated small EVs. Firstly, a spot count feature (Spot Count_Spot (M09, Ch09, Bright, 1, 1, 1) _4) was created to exclude any events with a brightfield image. A second spot count feature (M02, Emerald Green, Bright, 1, 5, 1) 4) was then created to include only the events with a single Emerald Green “dot” and to exclude any coincident or clumped EVs.

Assay controls

Following the MISEV 2018, 2023 and MIFlowCyt-EV framework for standardized reporting of EV flow cytometry experiments (Théry, et al. 2018, Welsh, et al. 2020, Welsh, et al. 2024), we employed various controls (Supplementary data, Table 1) to ensure reliable, specific detection and characterization of EVs. An unstained buffer control (PBS without dye or antibodies) was used to check for the background signal, while stained buffer control (PBS with ExoFlow-ONE™ dye) was used to identify false positives. The unstained EV control (EVs without dye or antibodies) was used to check for EV autofluorescence. Isotype controls (isotype antibodies with dye) were used to rule out the false-positive signals from the antibodies themselves. Lastly, size reference nanobeads (various- sized beads, fluorescent and non-fluorescent) were used.

Supplemental results

Enumeration and characterization of male reproductive tract EVs by IFC

Instrument calibration with correct reference materials is crucial for accurately aligning flow rate, light scatter, and fluorescence, thus ensuring that the EV signals are standardized, and data interpretation is simplified (Welsh, et al. 2024). We employed the ApogeeMix size reference (Apogee Flow Systems), aqueous silica (ɳ=1.43) and polystyrene (ɳ=1.59) bead mixture with diameters from 110-1300 nm as size references and guide the subsequent gating of small particles. The reference size nanobeads were first analysed (Supplementary Fig.1A), and gates were set to include all EVs <180 nm (Supplementary Fig. 1B) that stained positive with the ExoFlow-ONE™ EV labelling dye (Supplementary Fig.1C). This dye specifically labels membrane and internal EV components and offers superior specificity than traditional tetraspanin-based EV labelling owing to non-ubiquitous and non-uniform expression of tetraspanins on the surface of EVs (Coughlan, et al. 2020). The inbuilt 'Image Analysis' functionality of IDEAS ver. 6.2 software was employed to eliminate debris and coincident events, as previously explained (Gomez, et al. 2023). Larger particles were excluded by gating out frames containing visible brightfield spots (Supplementary Fig.1D). Only the frames displaying a single Emerald Green "dot" were selected to eliminate any coincident or clumped extracellular vesicles (EVs) by using the 'Spot Count' feature Supplementary Fig.1E). These gates were applied to the final plots, allowing for an accurate estimation of the collected statistics (Supplementary Fig.1F). The majority of MRT-EVs staining for the ExoFlow-ONE™ EV labelling dye were below the 180 nm gating threshold (Supplementary Fig.1A).

In accordance with the MISEV 2018, 2023 guidelines and the MIFlowCyt-EV framework for standardized reporting of EV flow cytometry experiments, the epididymosomes and seminal vesicle fluid EVs were stained with ExoFlow-ONE™ EV labelling dye (Supplementary Fig.2A-C) or no dye (EVs only, Supplementary Fig.2D-E), showing that the EVs were only present in the gate with ExoFlow-ONE™ dye-stained samples. A 'medium only' (1X PBS) control (Supplementary Fig.2G-I) and ExoFlow-ONE™ dye-stained buffer control (Supplementary Fig.2J-L) also showed no EVs. A significant difference between ExoFlow-ONE™ EV labelling dye-stained sample (EVs + Exo) and no dye (EVs Only, $P< 0.001$), stained buffer (Buffer + Exo, $P<0.001$), and buffer only ($P<0.001$) control groups (Supplementary Fig.3) for all three male reproductive tract EV subpopulations, indicating successful EV-specific labelling.

Initially, the male reproductive tract EVs s were stained only with the ExoFlow-ONE™ EV labelling dye and analysed. Next, these stained epididymosomes and Svf-EV samples were used to set the gates for CD9, CD63, and CD81 positivity (Supplementary Fig.4A) to establish the tetraspanin-positive MRT-EV population. The male reproductive tract EVs stained with ExoFlow-ONE™ EV labelling dye and anti-tetraspanin antibodies revealed that the epididymosome and seminal vesicle fluid EV samples had distinctive immunophenotypes exhibiting a differential abundance of CD9, CD63, or CD81 markers (Supplementary Fig.4B).

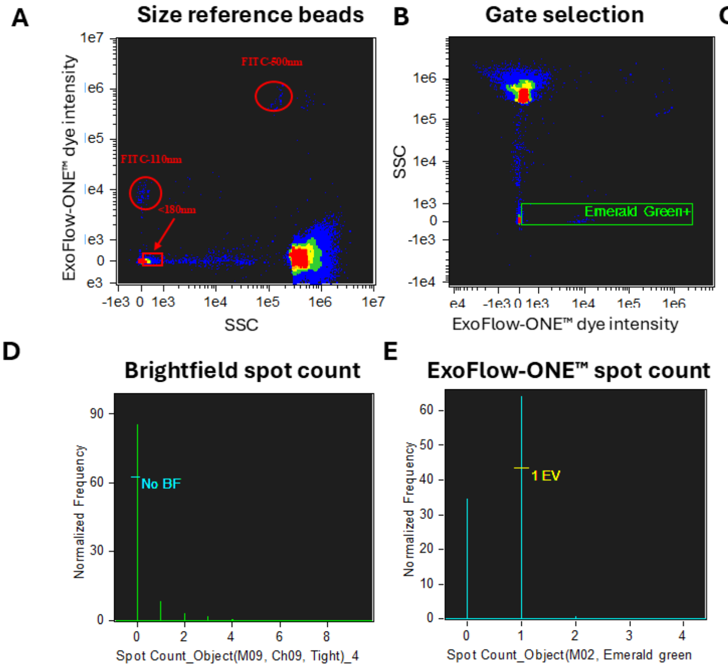

F **Statistics collected**

Population	Count	% Gated	Objects/mL
All	35388	100	222784969.51
Emerald Green+	6128	17.3	38578792.05

Supplementary Fig, 1: Size reference, gates and settings for characterization of male reproductive tract EVs with ExoFlow-ONE™ EV Labelling dye (Emerald Green) using imaging flow cytometry (IFC) To display the reference standard**,** first the (A) Reference size nanobeads (Apogee Flow Systems, 110-1300 nm) were analysed. (B) An EVs gate was then created below 180 nm representing exosomes and microvesicles (C) This gate was copied over to samples (seminal vesicle fluid EVs, representative image) collected for further gating using the ExoFlow-ONE™ EV labelling dye (Emerald Green). (D) To eliminate debris and coincident events, the Spot_count gate was set at 0 for Brightfield Channel 09; (E) The Spot_count for ExoFlow-ONE™ EV Labelling dye (Emerald Green) channel 02 was set at 1 for individual EV (<110 nm) events. (F) Statistics collected from the gated EV population in the male reproductive tract EV samples.

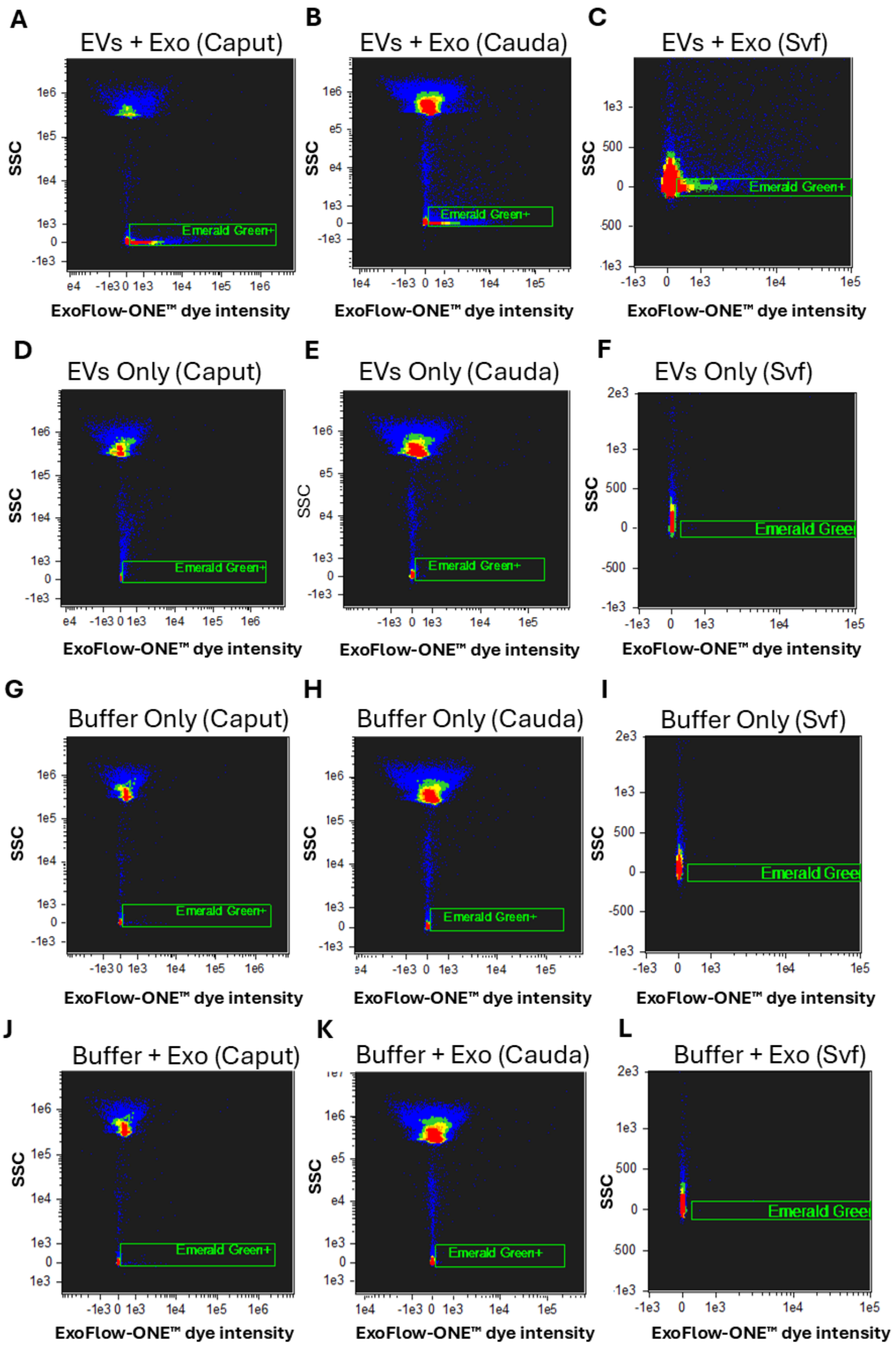

Supplementary Fig. 2: Characterization of male reproductive tract EVs using imaging flow cytometry and associated controls Representative plots for ExoFlow-ONE™ EV labelling dye-stained (A) caput and (B) cauda epididymosomes and (C) seminal vesicle fluid EV samples (EVs + Exo). Representative plots for unstained (D) caput and (E) cauda epididymosome and (F) seminal vesicle fluid EV samples (EVs Only). Representative plots for buffer (PBS) only sample control for (G) caput and (H) cauda epididymosomes and (I) seminal vesicle fluid EV samples (Buffer Only). Representative plots for ExoFlow-ONE™ EV labelling dye-stained media only sample (Buffer + Exo) controls for (J) caput and (K) cauda epididymosomes and (L) seminal vesicle fluid EV samples (Buffer Only).

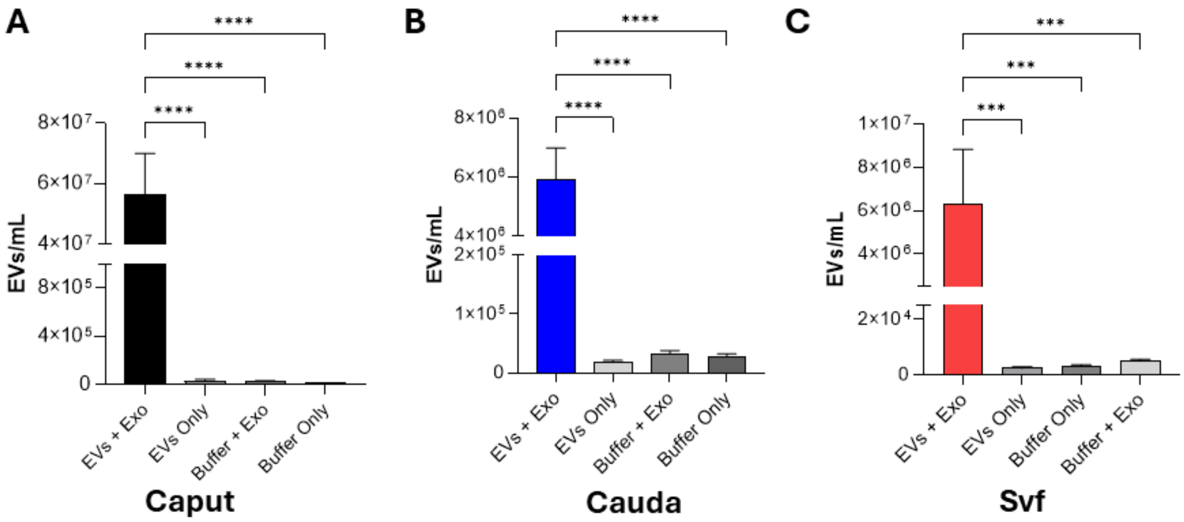

Supplementary Fig. 3: Finding EVs by ExoFlow-ONE™ EV labelling dye using imaging flow cytometry Significantly higher EVs/mL were observed in the ExoFlow-ONE™ EV Labelling dye-stained (D) caput, (E) cauda and (F) seminal vesicle fluid EV samples than the employed controls. Data are expressed as mean ± SEM. One-way ANOVA followed by Dunnett's post hoc analyses, *** $P<0.001$, **** $P<0.0001$.

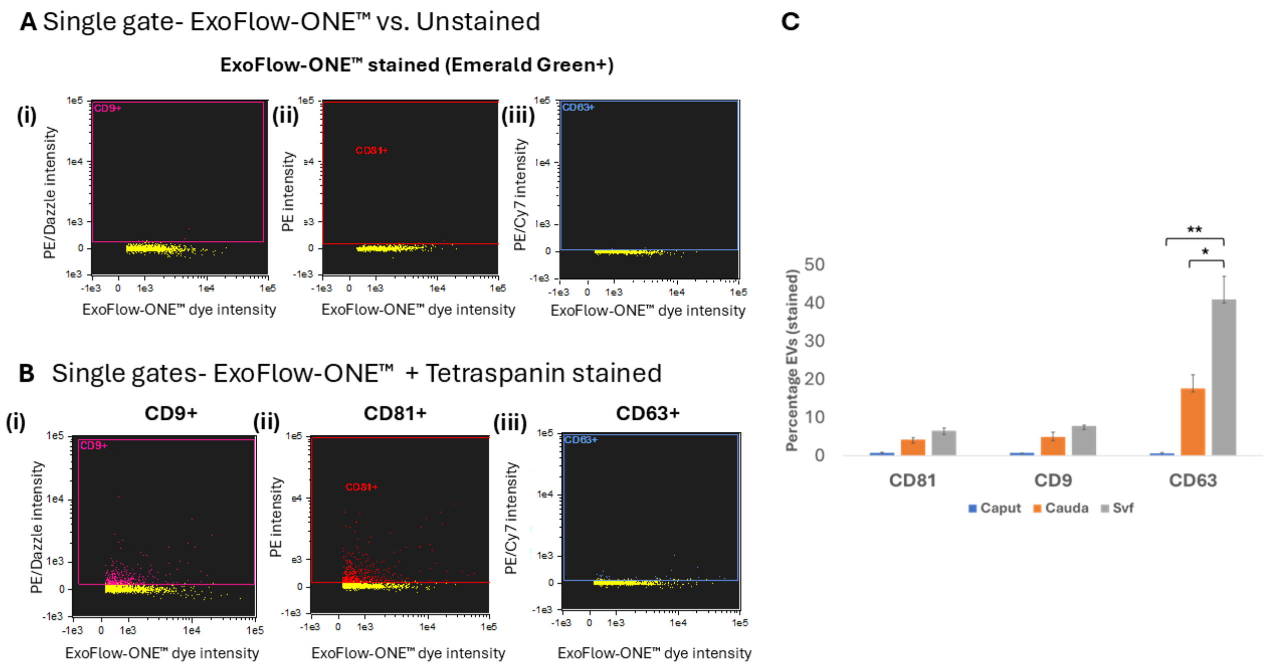

Supplementary Fig. 4: Immunophenotyping gating and controls Representative plots of (A) ExoFlow-ONE™ EV Labelling dye (Emerald Green) versus tetraspanins viz. (i) CD9; (ii) CD81; and (iii) CD63 using samples stained only with the ExoFlow-ONE™ EV Labelling dye (Emerald Green) to create gates for ExoFlow-ONE™ EV Labelling dye, tetraspanins and EVs. (B) Representative plots of ExoFlow-ONE™ EV labelling dye and tetraspanin stained male reproductive EVs for (i) CD9; (ii) CD81; (iii) CD63 (C) The expression of tetraspanin CD63 could only be validated in the caput epididymosomes. The data for CD63 profiling in the cauda epididymosomes and seminal vesicle fluid EVs was excluded because of the high binding of ISCs (>10%) (N.D. not determined). One-way ANOVA and unpaired two-tailed t-test, Tukey's post hoc analyses, **P<0.01 and *** P<0.001.
